# Frictional Behavior of Individual Vascular Smooth Muscle Cells Assessed By Lateral Force Microscopy

**DOI:** 10.3390/ma3094668

**Published:** 2010-09-14

**Authors:** Delphine Dean, Jason Hemmer, Alexey Vertegel, Martine LaBerge

**Affiliations:** Department of Bioengineering, Clemson University, 301 Rhodes Research Center, Clemson, SC, 29634, USA; E-Mails: finou@clemson.edu (D.D); jhemmer@clemson.edu (J.H.); vertege@clemson.edu (A.V.)

**Keywords:** atomic force microscopy (AFM), lateral force microscopy (LFM), friction, vascular smooth muscle cells

## Abstract

With the advancement of the field of biotribology, considerable interest has arisen in the study of cell and tissue frictional properties. From the perspective of medical device development, the frictional properties between a rigid surface and underlying cells and tissues are of a particular clinical interest. As with many bearing surfaces, it is likely that contact asperities exist at the size scale of single cells and below. Thus, a technique to measure cellular frictional properties directly would be beneficial from both a clinical and a basic science perspective. In the current study, an atomic force microscope (AFM) with a 5 µm diameter borosilicate spherical probe simulating endovascular metallic stent asperities was used to characterize the surface frictional properties of vascular smooth muscle cells (VSMCs) in contact with a metallic endovascular stent. Various treatments were used to alter cell structure, in order to better understand the cellular components and mechanisms responsible for governing frictional properties. The frictional coefficient of the probe on VSMCs was found to be approximately 0.06. This frictional coefficient was significantly affected by cellular crosslinking and cytoskeletal depolymerization agents. These results demonstrate that AFM-based lateral force microscopy is a valuable technique to assess the friction properties of individual single cells on the micro-scale.

## 1. Introduction

While significant steps have been made towards understanding the bulk mechanical properties of living cells, comparatively little is known about their surface frictional properties. The study of cellular frictional properties is of interest for a variety of reasons. For example, numerous physiological processes including blood flow [1], articulating cartilaginous tissues [2], respiration [3], cell adhesion [4], and cell migration [5], are all affected in some manner by cellular and tissue frictional properties. With regard to endovascular surgical procedures, increased knowledge of cellular frictional properties could also be of a significant clinical value. The deployment of endovascular devices results in the exertion of mechanical shear forces on underlying vascular endothelial and vascular smooth muscle cells (VSMCs) [6]. In cases of complex or tortuous vascular lesions, a reduction of friction has been shown to ease delivery of endovascular devices [7]. The process of stent placement typically results in endothelial denudation [8]. As a result, stent struts are in direct contact with underlying VSMCs. More accurate computer modeling of *in vivo* stent behavior could be accomplished if the frictional properties of vascular cells were elucidated [6,9]. Similarly, finite element models of atomic force microscope (AFM) cell mechanics experiments have thus far relied on assumptions of frictional conditions, due to a lack of experimental data [10,11]. 

Numerous cellular constituents may play a role in governing frictional properties. The glycocalyx, an extracellular matrix of proteglycans and glycoproteins is believed to play a role in the lubrication of red blood cells [1] and endothelial cells [12]. Additionally, the glycocalyx is involved in mechanical signal transduction in both endothelial cells and VSMCs [13]. In the case of VSMCs, the glycocalyx is composed primarily of chondroitin sulfate and heparan sulfate [14]. Located beneath the glycocalyx, the cell membrane is composed of a lipid bilayer containing a wide array of transmembrane proteins. Lipid bilayers alone have complex frictional and viscous properties [15], and in the case of intact living cells, these properties are likely made even more complex by the presence of transmembrane molecules and surface charge distribution. Finite element research on the frictional properties of soft biological tissues has shown a positive correlation between the friction coefficient and the modulus of elasticity [3]. It is quite possible that this same relationship would exist for individual cells, in which case the cytoskeleton, the main determinant of cellular elastic modulus, would also play a significant role in governing cellular frictional properties. In many cell types, including VSMCs, actin and microtubules are the cytoskeletal components primarily responsible for determining cellular mechanical properties [16,17,18]. All of these cellular constituents (glycocalyx, cell membrane, cytoskeleton) are of course physically linked with one another, making it rather difficult to fully separate the effects of each on whole-cell frictional properties.

The atomic force microscope (AFM) is a useful tool for the measurement of frictional forces at the nano- and micro- scales [19], a technique often referred to as lateral force microscopy (LFM). Numerous studies have examined frictional properties on a range of materials, including various polymers, films, and lipid bilayers [19,20]. Currently however, very little data exist relating to the frictional properties of living cells or tissues at the micro-scale. However, recent studies have examined macro-scale frictional properties of vascular endothelial cells [6] and corneal epithelial cells [21,22]. The AFM technique described herein is capable of providing microscale frictional data to complement the available macroscale data. A significant advantage of AFM-based cellular friction measurements is the use of considerably lower normal loading forces as compared to macro-scale, reducing the chances of any cell damage occurring during the experimental procedure. 

The main goal of the current study was the development of an AFM-based technique for micro-scale measurement of individual cell surface frictional properties, as well as elucidation of the cellular physical constituents responsible for governing frictional behavior. Vascular smooth muscle cells were chosen for this research due to the clinical relevance of their frictional properties with respect to endovascular surgical procedures.

## 2. Results and Discussion

### 2.1. Results

**Figure 1:**
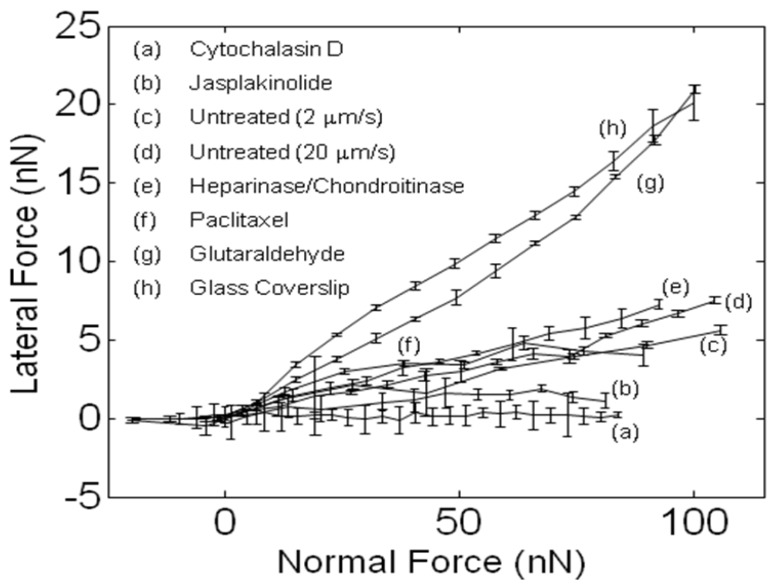
Representative frictional data (lateral force *vs.* normal force) from each VSMC group. Each curve corresponds to an individual cell chosen as a representative example due to its proximity to its respective VSMC group mean frictional coefficient. Each data point within a curve represents the mean ± SD for a particular normal force.

Calibration of the AFM cantilevers were performed as described in the Methods section. For the cantilevers used in these experiments, the lateral sensitivity, α, was found to be approximately 68 nN/V. Following calibration, the cantilevers were used to perform AFM lateral force measurements on the surface of individual VSMCs (see Methods section of details). In total, 16 data points were collected at each given normal force, for each individual cell. The resultant average lateral force *vs.* normal force for the borosilicate spherical probe against a representative cell from each group is shown in Figure 1. Cells were in one of four groups based on the treatment they received prior to the AFM testing: untreated control, fixation with glutaraldehyde, incubation with chondroitinase and heparinase, incubation with cytochalasin D, incubation with Jasplakinolide, and incubation with Paclitaxel. Each of these agents was chosen due to their known effects on cellular structure. Glutaraldehyde is a commonly used fixative for cells and tissues that induces significant crosslinking. Cytochalasin D is an agent known to disrupt the actin network of living cells. Jasplakinolide stabilizes actin fibers and Paclitaxel stabilizes microtubules. Chondroitinase and heparinase are responsible for the enzymatic degradation of the constituents of the glycocalyx. 

**Figure 2:**
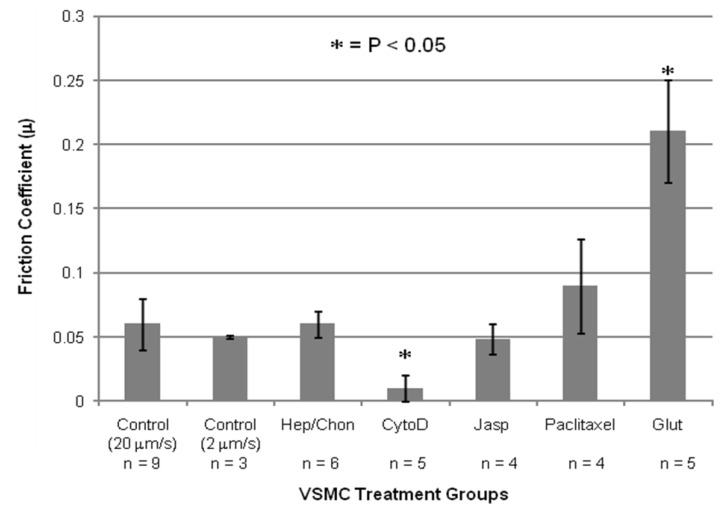
VSMC coefficients of friction. Data are presented as mean ±SD. Control untreated cells were tested at 20 μm/s and 2 μm/s scanning speeds. The treatment groups were: Hep/Chon = heparinase/chondroitinase treatment, CytoD = cytochalasin D treatment, Jasp = jasplakinolide treatment, Paclitaxel treatment, and Glut = Glutaraldehyde treatment.

One-way ANOVA comparison of friction coefficients indicated a statistically significant difference between groups, p < 0.001. Subsequent pair-wise comparisons indicated significantly greater friction coefficients for glutaraldehyde treated VSMCs (µ = 0.21 ± 0.04) *vs.* controls (µ = 0.06 ± 0.02), p = 7.8 × 10^−6^, and significantly lower friction coefficients for cytochalasin D treated VSMCs (µ = 0.01 ± 0.01) *vs.* controls, p = 3.4 × 10^−6^. No significant difference was found between chondroitinase/heparinase treated VSMCs (µ = 0.06 ± 0.01) and controls (p = 0.41). There were no significant difference in friction coefficients for Jasplakinolide treated VSMCs (μ = 0.048 ± 0.01) and controls (p = 0.31) Paclitaxel treatment resulted in a small increase in friction coefficient (μ = 0.089 ± 0.04) compared to controls (p = 0.058). Untreated VSMCs were scanned at different speeds to determine if scanning speed had any effect on the measured friction coefficient. There was no significant difference between untreated VSMCs measured at 20 or 2 µm/s (µ = 0.05 ± 0.001), p = 0.17. As a further control, the friction coefficient between the borosilicate tip and a bare glass coverslip surface was measured before and after cell testing. The mean coefficient of friction of the borosilicate spherical probe on the glass coverslips in media was 0.18 ± 0.09, which is in the range of the coefficient of friction for glass on glass under lubricated conditions (0.1 to 0.6) [23]. In addition, there were no significant differences in the measured glass-glass friction coefficient taken before and after cell testing. This suggests that the cell friction measurements did not significantly change the probe tip surface through additional biofouling. Measured coefficient of friction values for all VSMC groups are compared in Figure 2.

**Figure 3:**
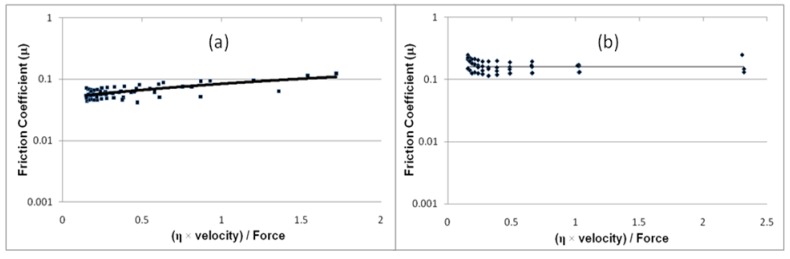
(**a**) Coefficient of friction for untreated VSMCs plotted versus an approximated Sommerfeld number. The data points are from 5 separate VSMCs, and were fit with a linear trendline, R^2^ = 0.539; (**b**) Coefficient of friction for glutaraldehyde-treated VSMCs plotted versus an approximated Sommerfeld number. The data points are from 5 separate VSMCs, and were fit with a linear trendline, R^2^ = 5 × 10^–5^.

To understand the characteristics of the frictional behavior of the VSMCs, Stribeck curves were plotted for the control and gluteraldehyde-treated cell groups. A Stribeck curve is used in tribology to visualize how friction relates to viscosity, velocity, and load by plotting the friction coefficient versus the Sommerfeld bearing characteristic number. An approximated Stribeck curve was plotted using data from 5 separate cells for each condition, with an approximation of the Sommerfeld number calculated as:

(η × v)/F_N_

where η is the dynamic viscosity of DMEM (0.00078 Pa·s)[24], v is probe velocity (20 µm/s), and F_N _is normal force (N). A linear fit to data in the Stribeck curve for untreated VSMCs, revealed a positive slope with increasing (η × v)/F_N_ ratio (Figure 3a). In contrast, the Stribeck curve for glutaraldehyde-treated VSMCs revealed a relatively flat relationship between friction coefficient and (η × v)/F_N_ (Figure 3b). A Stribeck curve for the Cytochalasin D treated cells was not calculated as the cytochalasin D treated VSMCs underwent a noticeable bulk reciprocating motion in phase with probe motion during testing.

### 2.2. Discussion

The principal goal of the current study was to investigate the development of an AFM-based method for measurement of cell surface frictional properties on the micro-scale, with a specific interest in vascular smooth muscle cells. Secondly, an attempt was made to elucidate which cellular structural components are responsible for governing frictional behavior. To the best of the authors’ knowledge, the current data represent the first reporting of cellular surface frictional coefficients obtained via AFM. 

The mean coefficient of friction for untreated VSMCs found in the current study (0.06 ± 0.02) is similar to macroscale values previously reported for endothelial cells (*μ* = 0.03 – 0.06) [6] and corneal epithelial cells (*μ* = 0.05 ± 0.02) [22]. It should be noted that not only were these previous cellular friction studies conducted on different cell types on the macroscale, but probe speed, diameter, and velocity were all substantially different than the current study. Each of these studies was carried out in media with 10% serum, meaning there was undoubtedly significant protein adhesion to the probe. Such protein adhesion however, is precisely what one would expect from almost any implant material once it comes in contact with blood. Protein adsorption has been found to increase friction on polymer surfaces [25], and it may very well have had an effect under the current conditions, although this was not investigated. No detachment or noticeable displacement of cells was observed in any of the VSMC sample groups during friction testing, indicating that the cells were firmly adhered to the substrate. The lone exception to this was the observation that cytochalasin D treated VSMCs underwent a noticeable bulk reciprocating motion in phase with probe motion. Through optical microscopy observation, cells did not appear to be physically damaged as a result of AFM probe contact. Furthermore, the use of spherical as opposed to pyramidal AFM probes, greatly reduces contact stresses at a given normal force and lessens the chances of cell damage [26]. 

In previous studies using AFM indentation and stress-relaxation measurements to assess VSMC mechanical properties [18], cytochalasin D was found to significantly reduce VSMC modulus, Paclitaxel was found to cause a small increase on VSMC modulus, and Jasplakinolide showed no effect on cell modulus. In addition, glutaraldehyde crosslinked cells are significantly stiffer than controls. In this study, Glutaraldehyde (a crossklinking agent) and cytochalasin D (an actin depolymerizing agent) caused significant increases and decreases in frictional coefficients, respectively. Paclitaxel, a microtubule stabilizing agent, caused a small increase in frictional coefficient while Jasplakinolide, an actin stabilizing agent, showed no effect. Taken together, these observations serves as evidence that bulk cellular modulus does correlate positively with frictional coefficient, as it does with whole tissue [3]. Although VSMC exposure to the glycocalyx degrading enzymes for chondroitin sulfate and heparan sulfate did not significantly affect frictional coefficients in the current study, this does not mean that the glycocalyx should be ruled out as a source of VSMC lubrication. It is very difficult to fully digest the glycocalyx without killing the cells. Although they have previously been shown to influence VSMC shear stress-mediated contractile response, the concentrations of enzymes used in this study only partially remove the glycolalyx [13]. Higher enzyme concentrations resulted in cell death and thus, cell friction coefficients could not be measured. Interestingly, it should be noted that the measured coefficient of friction of the control cells in this study (0.06 ± 0.02) is comparable to the friction coefficient measured previously between a similar spherical AFM probe and surface coated with aggrecan, a chondrotin sulfate-rich proteoglycan, in 0.1 M NaCl solution (0.06 ± 0.01)[20]. Consequently, further investigation is warranted. It has been suggested that fluid film lubrication, and in particular elastohydrodynamic lubrication, is a common lubricating mechanism in biological systems [27]. Certainly, vascular smooth muscle cells such as those used in the current study are soft enough to be subject to deformation under most loading regimes. However, given that fluid film coefficients of friction are typically in the range of 0.001 to 0.01, while boundary lubrication yields friction coefficients closer to 0.1, it is also possible that the intermediate values found for untreated VSMCs are indicative of a mixed lubrication regime [28] for the contact and tribological conditions used in this study. The Stribeck curve for untreated VSMCs plotted in the current study revealed a positive correlation between frictional coefficient and the approximated Sommerfeld number. This behavior is representative of fluid film lubrication. This is in agreement with previously a published finite element simulation of elastohydrodynamic lubrication, showing decreasing frictional coefficients with increased pressure in soft biological tissues [3]. It is possible that glutaraldehyde-fixed VSMCs exhibited a different lubrication profile either due to mechanical changes (increased stiffness) or chemical changes at the cell surface due to cross-linking of cell components. If the former cause is true, and VSMC mechanical and frictional properties also varied *in vivo*, this could result in intrinsic variable contact stresses within the tissue, and a variable frictional profile over the surface, leading to disturbed contact stresses [29]. 

Future mechanistic studies examining VSMC lubrication regimes are indeed warranted, especially to further determine the effects of velocity, load, and lubricant viscosity. One potential benefit of an AFM-based cellular friction technique is the ability to modify the AFM probes with any number of different surface chemistries, for instance based on charge or hydrophilicity. In combination with treatments altering the physical chemistry and makeup of the underlying cells, such experiments could help reveal the underlying mechanisms of cellular and tissue lubrication. The technique described in the current study could be used to gather valuable information in the area of biotribology. Given the mechanical and material complexity of living cells, innovative and accurate frictional measurement techniques will be needed to determine the underlying mechanism(s) of friction and lubrication.

## 3. Experimental Section 

### 3.1. Cell Culture

Rat aortic VSMCs were isolated from adult male Sprague-Dawley rats and cultured in Dulbecco’s Modified Eagle’s Medium (DMEM) (Mediatech, Herndon, VA, USA) with fetal bovine serum (FBS) (10%) (Sigma, St. Louis, MO, USA) and antibiotic/antimycotic solution (1%) (Sigma). Cells were maintained in T-75 polystyrene flasks in an incubator at 37 °C, with 5% CO_2_, and fresh media was exchanged every 48 hours. Cells were allowed to grow to ~80% confluency in the flasks, then trypsinized with 0.25% trypsin (Mediatech) / 0.02% EDTA (Sigma) and seeded onto 22 × 22 mm glass coverslips (VWR, West Chester, PA, USA) at a density of 150,000 cells per coverslip Seeded coverslips were incubated in 6-well plates with DMEM (10% FBS) at 37 °C with 5% CO_2_, and media was exchanged every 48 hours prior to AFM experimentation. Cells were utilized between passages 5 and 8.

### 3.2. AFM Friction Experiments

**Figure 4:**
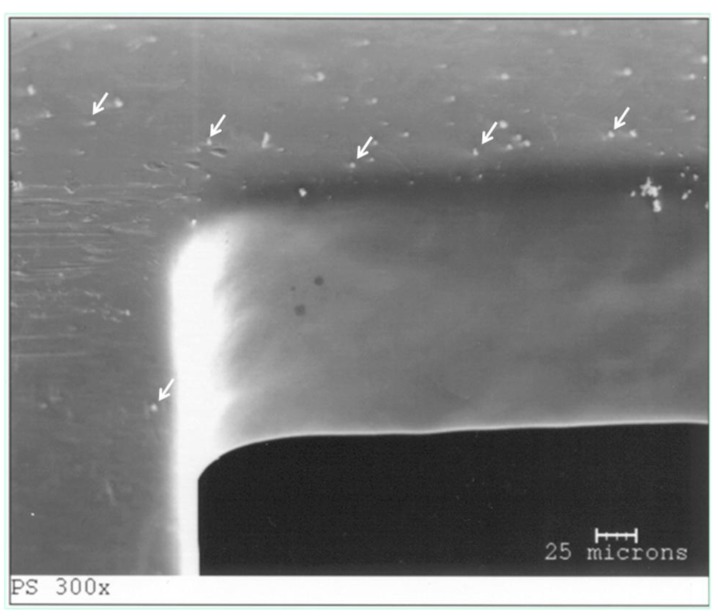
Scanning electron micrograph of a stainless steel stent, illustrating size and prevalence of surface asperities (six example asperities are marked with white arrows).

Atomic force microscopy (AFM) experiments were performed once cells had formed a confluent monolayer on coverslips, typically between 5 to 6 days. Confluent cells were used as they more accurately replicate the high cell densities found in vivo [30]. In addition, confluent cells were used in previous macroscale measures of vascular cell shear and friction response experiments [6,30]. For all AFM experiments, a Veeco Dimension 3000 AFM with a Nanoscope IV controller (Veeco Metrology, Santa Barbara, CA, USA) was operated in contact scanning mode. A borosilicate spherical-tipped AFM probe (5 μm diameter) (NovaScan, Ames, IA, USA) on a silicon-nitride cantilever with a nominal spring constant of 0.12 N/m, was used throughout the study. A 5 µm AFM probe was chosen, in part, due to its proximity in size to that of asperities previously observed on the surface of endovascular stents in our laboratory (Figure 4). During AFM experiments, cells were kept on the coverslips in their culture media (DMEM w/ 10% FBS). To maintain media temperature, experiments were either performed with a custom heat stage or the media was exchanged approximately every 30 minutes with warm 37 °C media throughout the course of the AFM experiments, each lasting for approximately 1 h in total. There were no statistical differences between data collected while using the heating stage or those collected with frequent media changes.

Cells received one of the following treatments prior to AFM friction experiments; fixation with 3% glutaraldehyde overnight at room temperature, incubation with 0.2 U/ml of chondroitinase ABC and 0.2 U/ml heparinase III (Sigma, St. Louis, MO, USA) for 30 min. at 37 °C, incubation with 5 µM cytochalasin D (Sigma) for 30 minutes at 37 °C, incubation with 0.1 μM Jasplakinolide for 1 hr at 37 °C, or incubation with 10 μM Paclitaxel for 1 h at 37 °C. Each of these agents was chosen due to their known effects on cellular structure. Glutaraldehyde is a commonly used fixative for cells and tissues that induces significant crosslinking. Cytochalasin D is an agent known to disrupt the actin network of living cells. Jasplakinolide helps to stabilize actin filaments while Paclitaxel stabilizes microtubules. In addition, Paclitaxel is interesting since it is used in some drug-eluting stents to prevent VSMC hyperplasia. The concentration and incubation times were selected based on our previous studies [18] and those of other groups [31,32]. Chondroitinase ABC and heparinase III are responsible for the enzymatic degradation of the glycocalyx constituents chondroitin sulfate and heparan sulfate, respectively. Untreated VSMCs were used as controls. 

**Figure 5:**
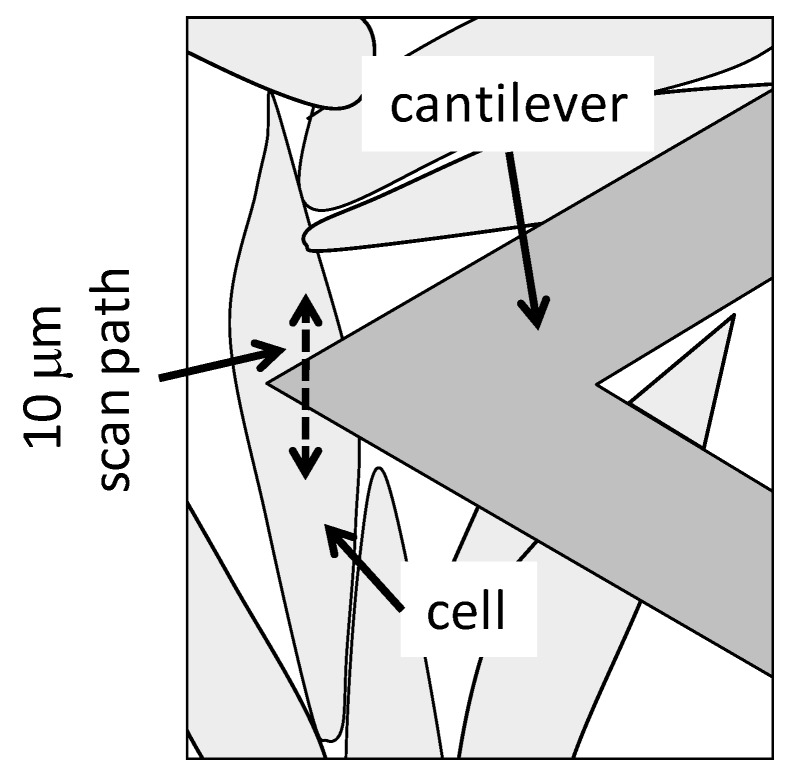
Schematic of the experimental setup. The AFM probe positioned over a single VSMC, with a 10 μm reciprocation path along the major axis of the cell. VSMC in the experiment were about 10 μm wide and 20–40 μm long.

**Figure 6:**
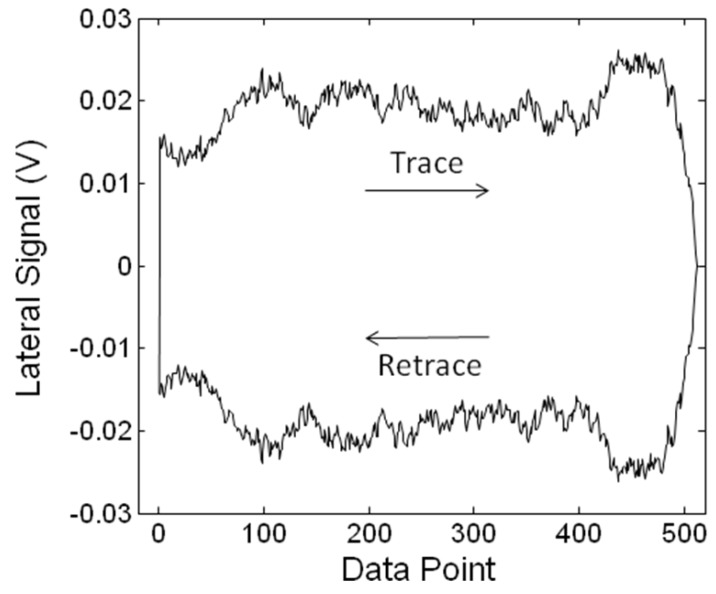
Representative lateral signal trace and retrace curves from a control VSMC at ~8 nN applied normal force. 512 data points were collected on each line. Recording trace and retrace data allows one to distinguish frictional from topographical effects on the lateral signal [19]; The friction signal (in V) is calculated as half the difference of the trace and retrace signals.

Following the identification of a target cell, the AFM probe was positioned over the central region of the cell body. Elongated cells were chosen for friction testing, with the AFM probe reciprocating along the major axis of each test cell as in Figure 5, thus preventing the probe from coming into contact with adjacent cells or substrate. That is, cells were selected for testing based on their orientation relative to the tip. Their major axis was in the direction of the AFM scan so as to ensure that the tip would remain on that single cell during the 10 μm scan necessary for the friction measurement. The sample was rotated between measurements so as to ensure that the average data included cells of all different orientation. As standard in the lateral (friction) microscopy mode of AFM, the sample was scanned at a 90° scan angle and the lateral trace and retrace signals were recorded (Figure 5 and Figure 6) [19,20,33,34]. The scan size (reciprocating cutoff length) was 10 µm.

Most measurements were performed at tip velocity of 20 µm/s, However, for experiments on control cells, a velocity of 2 µm/s was also used in order to evaluate velocity-dependence. Normal forces were incrementally increased by adjusting the deflection setpoint from approximately –2 to +8 volts, in 0.25 to 0.5 volt increments. Since the setpoint was set to –2 volts prior to engaging and given the normal deflection sensistivity and spring constant of the cantilevers, these setpoint values translate to a range of approximately 0 to 100 nN of normal force applied during the experiments. For each deflection setpoint on a given cell, 16 lines of trace and retrace frictional data were recorded, with 512 data points recorded per line (Figure 6). Therefore, since the image aspect ratio was 32 and the scan path length was 10 μm, the final scan size covered a 10 μm by 0.3125 μm area on the cell surface.

The normal deflection sensitivity and spring constant were measured using standard methods [35]. The normal spring constant for each cantilever was determined from measurements of cantilever thermal fluctuations (“thermal tune” method for cantilever spring constant calibration [35,36]). The deflection (or photosensor) sensitivity (nm/V) was obtained from the slope of the contact portion of force curves obtained on control glass slides [35,37]. 

The maximum normal force exerted on each cell was in the range of 100 nN, with the exact value dependent upon deflection sensitivity and cantilever spring constant. At the start and finish of each experiment, frictional force data were also collected on a clean glass coverslip in media. The resulting friction coefficients could then be compared to existing data on the coefficient of friction between 2 glass surfaces under lubricated conditions, revealing whether the values determined for cells were reasonably accurate. 

The lateral sensitivity of the cantilever was determined using the previously published “modified wedge method” for calibration of micro-sized tips [34,38]. To ensure that it remained consistent during testing, the lateral sensitivity was measured both before and after the experiment. Briefly, the probe was used to collect frictional force data on a flat mica surface, as well as a mica surface positioned at a known angle of incline. The resulting voltage *vs.* normal force plots are used to determine the lateral sensitivity, α, of the cantilever. This lateral sensitivity value is then used to convert all lateral deflection signals (V) into lateral force values (nN). Cell surface frictional coefficients (µ) were then calculated based on the equation:

F_L_ = µ × F_N_

where F_L _is the lateral force acting on the AFM probe, and F_N _is the normal force. The normal force applied at each deflection setpoint was determined by the product of the deflection setpoint (V), deflection sensitivity (nm/V), and cantilever spring constant (N/m). 

### 3.3. Data Analysis

Following each experiment, data were processed using custom MATLAB (The Mathworks, Inc., Natick, MA, USA) scripts. Excessive noise was filtered from the data by eliminating all data points greater than 2 standard deviations away from the mean lateral signal value at a given normal force. The net lateral signal from each pair of trace and retrace lines is calculated as half of the difference between the two of them. Laser drift that occurred during frictional measurements was accounted for by recording the lateral voltage on the photodiode at the start and finish of each cell, and correcting using a linear algebraic function. Mean lateral forces and standard deviations were plotted *vs.* normal forces, from which a linear fit was used to determine the slope of each line, *i.e*., the coefficient of friction, µ. 

### 3.4. Statistical Analysis

Coefficients of friction between groups were compared using one-way analysis of variance (ANOVA) tests with subsequent pairwise comparison using Students t-tests. All tests were performed with an alpha of 0.05, and p-values less than 0.05 were considered statistically significant.

## 4. Conclusions

Measurement of cellular surface frictional properties via AFM is a viable, albeit complex technique. In the current study, frictional coefficients of untreated VSMCs were found to be approximately 0.06. Frictional coefficients were increased by cellular crosslinking, and decreased by cytoskeletal depolymerization. Further study using this technique is needed to determine the precise mechanisms underlying cellular lubrication. 
